# Markers of liver regeneration—the role of growth factors and cytokines: a systematic review

**DOI:** 10.1186/s12893-019-0664-8

**Published:** 2020-02-12

**Authors:** Katrin Hoffmann, Alexander Johannes Nagel, Kazukata Tanabe, Juri Fuchs, Karolin Dehlke, Omid Ghamarnejad, Anastasia Lemekhova, Arianeb Mehrabi

**Affiliations:** Department of General, Visceral and Transplant Surgery, Ruprecht Karls University, Im Neuenheimer Feld, 110 69120 Heidelberg, Germany

**Keywords:** Liver regeneration, Biochemical markers, Post-hepatectomy liver failure, Cytokines, Growth factors

## Abstract

**Background:**

Post-hepatectomy liver failure contributes significantly to postoperative mortality after liver resection. The prediction of the individual risk for liver failure is challenging. This review aimed to provide an overview of cytokine and growth factor triggered signaling pathways involved in liver regeneration after resection.

**Methods:**

MEDLINE and Cochrane databases were searched without language restrictions for articles from the time of inception of the databases till March 2019. All studies with comparative data on the effect of cytokines and growth factors on liver regeneration in animals and humans were included.

**Results:**

Overall 3.353 articles comprising 40 studies involving 1.498 patients and 101 animal studies were identified and met the inclusion criteria. All included trials on humans were retrospective cohort/observational studies. There was substantial heterogeneity across all included studies with respect to the analyzed cytokines and growth factors and the described endpoints.

**Conclusion:**

High-level evidence on serial measurements of growth factors and cytokines in blood samples used to predict liver regeneration after resection is still lacking. To address the heterogeneity of patients and potential markers, high throughput serial analyses may offer a method to predict an individual’s regenerative potential in the future.

## Introduction

Post-hepatectomy liver failure (PHLF) is a serious complication after liver resection and the incidence varies from 1.2 to 32% [1–4]. PHLF is defined as functional deterioration of the liver associated with an increased international normalized ratio (INR) and hyperbilirubinemia on, or after, the fifth postoperative day [1]. There are recommendations that PHLF could be prevented if the future liver remnant (FLR) is not smaller than 20% of the original liver size in patients with normal liver function and 30–40% in patients with steatohepatitis or cirrhosis [5, 6]. Nevertheless, even with adequate pre-operative assessments and careful indications, PHLF is a major contributor to mortality rates of up to 5% after liver resection [7, 8]. Various patient- (comorbidities, age, and previous chemotherapy), parenchyma- (cirrhosis, fibrosis, cholestasis, and steatosis), and surgery-related factors (extent of resection, blood loss, and ischemia reperfusion injury) affect the regenerative capacity of the FLR [9, 10]. However, to predict the adequate size and individual regenerative capacity of the FLR remains a significant challenge for clinicians, surgeons, and scientists. The current PHLF therapy focuses on symptomatic and supportive treatment of the progredient dysregulation in the hepato-organic axis. However, the ultima ratio for PHLF is liver transplantation if patients fulfill listing regulations. This poses a marked morbidity and mortality risk for patients, and surgeons and clinicians should aim to ensure that postoperative liver failure does not occur. In clinical practice, there is a high variety of morphological and biochemical assessment methods for qualitative (indocyanine green retention rates; LiMAx-tests, MELD or CHILD-PUGH scores) and quantitative (computed tomography liver volumetry, analysis of bilirubin, transaminases, albumin) predictions for liver function in the context of liver resection [11]. However, non-invasive individualized identification of valid predictive and prognostic biomarkers of PHLF based on the cytokines and hepatic growth factors in the liquid-biopsy samples might be a novel approach in the peri-operative diagnosis and monitoring of regeneration on a molecular basis. The growing subgroup of high-risk patients with hepatic steatosis, steatohepatitis, or sinusoidal obstruction syndrome, after neo-adjuvant chemotherapy, in particular, would benefit from markers that indicate the livers’ individual abilities to cope with extended surgical resection [12]. Since liver regeneration is a well-orchestrated process controlled by various cytokines and growth factors, these might also be promising targets for modulation. Despite the growing knowledge of regeneration-associated signaling pathways and regulatory mediators in rodents, conversion of the process into humans and clinical practice has just begun [13].

Therefore, the purpose of this review was to systematically summarize current evidence on the cytokine- and growth factor- mediated signaling pathways in liver regeneration for the benefit of clinicians and surgeons, and to discuss their suitability for individual mediator-based regeneration predictions in patients.

## Methods

Protocol and registration: there was no review protocol and the study was not registered.

Eligibility criteria: inclusion of the studies was based on the Population, Intervention, Comparison, Outcome and Study design (PICOS) strategy with the following inclusion criteria [14]:

- Population: all patients undergoing liver resection
- Intervention: reports of measurements of cytokines and growth factors in the context of PHLF
- Comparator: no measurements of cytokines and growth factors,
- Outcome: association with PHLF
- Study design: any study except study protocols, letters, and common overviews.

Report characteristics: There were no restrictions regarding languages, years of publication, or publication status in the initial search. Original articles, case reports, clinical trials, reviews, meta-analyses, and systematic reviews were all included. In addition, reference lists of relevant articles and reviews were crosschecked for additional studies. Non-peer reviewed studies were excluded.

Information sources: The MEDLINE and Cochrane Library databases were searched for relevant studies; last search was conducted in April 2019.

Search: Search strategies included the following Medical Subject Headings (MeSH) in various combinations: liver regeneration, liver resection, partial hepatectomy, major liver resection, hemi-hepatectomy, post-hepatectomy liver failure, cytokine, growth factor, hepatocyte growth factor (HGF), tumor necrosis factor alpha (TNF-α), interleukin 6, epidermal growth factor (EGF), insulin-like growth factor (IGF), vascular endothelial growth factor (VEGF), fibroblast growth factors (FGFs), angiopoietin, platelet-derived growth factor (PDGF), proliferating cell nuclear antigen (PCNA), Ki-67, and micro-RNA (miRNA).

**Figure 1:**
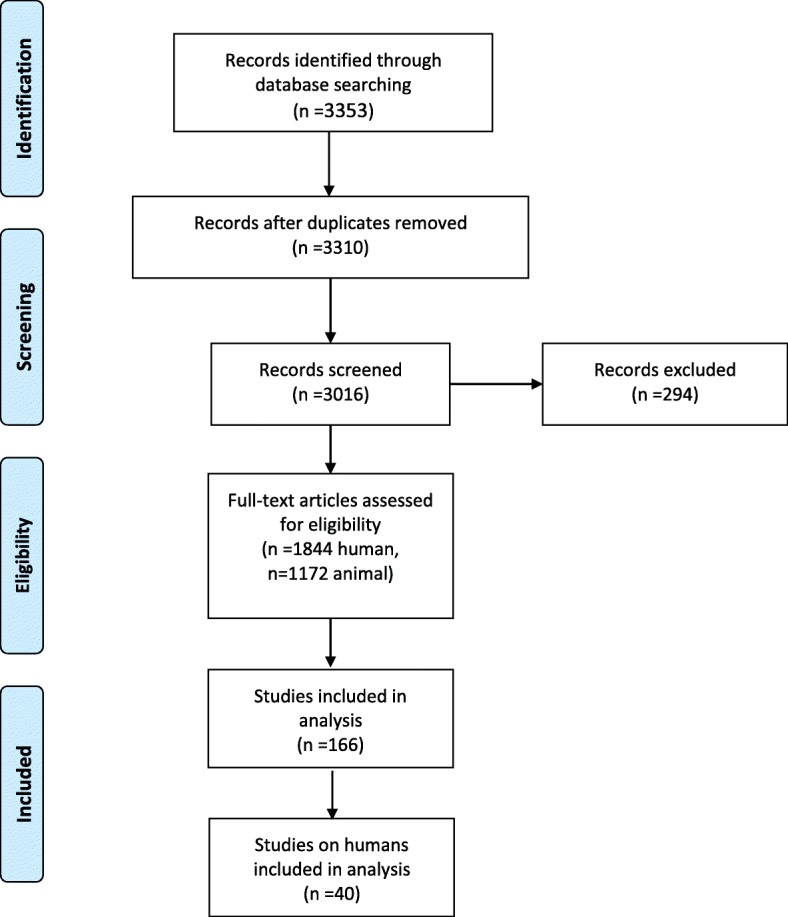
**Fig. 1** Study selection process

Study selection: Two authors (AN and YT) independently screened the titles and abstracts of all retrieved references and obtained full-text articles in cases of potential eligibility. Full texts of all animal studies and studies including patients that provided data on cytokine- and growth factor- mediated regeneration processes were analyzed according to the eligibility criteria. A third author (KH) was consulted in case of disagreement. Three thousand three hundred fifty- three articles were identified. After excluding duplicates (*n* = 294) and non-English studies (*n* = 43), 1172 animal studies and 1844 human studies were analyzed. Ultimately, 40 studies including 1498 patients were included in this review (Fig. 1). Studies were included based on predefined selection criteria: relevant information regarding measurements of available markers; clearly defined outcome parameters (such as PHLF according to International Study Group of Liver Surgery definitions); regeneration measured by clinically relevant methods such as computerized tomography scans, magnetic resonance imaging, or well-established laboratory methods such as cytology including any standard staining techniques (i.e. hematoxylin and eosin, Papanicolaou); molecular detection methods (with or without immunocytochemistry); any form of reverse-transcriptase polymerase chain reaction ([RT]-PCR) tests; and protein analyses which may include Western Blots or Fluorescence-activated cell sorting.

Studies were excluded if the language was not English, not published in peer-reviewed journals, and if the above-mentioned definitions of cytology or molecular diagnostics were not met. However, human trials included no randomized controlled trials, no multi-center trials, 37 prospective single-center trials, and 3 retrospective analyses. Case numbers were < 50 in the majority of trials.

Data collection process: Data extraction from reports was performed in duplicate using excel files. Due to the narrative character of the reviews and the analyses of animal as well as human studies, data were extracted comprehensively. The following data were extracted from every article: first author, year of publication, study type, enrollment period, sample size, definition of regeneration, incidence of PHLF, timing of detection, the detection protocol, target proteins, genes and antigens, reported outcomes, and the use of multivariate models.

Risk of bias in individual studies: Since no clinical endpoint was evaluated, these studies were not assessed for risk of bias according to Methodological Index for Non-Randomized Studies criteria [15].

## Results

### Temporal sequence of regeneration

On a cellular level, regeneration after resection consists of a compensatory hypertrophy followed by hyperplasia of the remaining hepatocytes. Three distinctive phases describe this phenomenon: initiation (0–5 h after resection), proliferative (5–144 h), and termination [16]. The injury inflicted by hepatic resection triggers a signaling cascade that mobilizes immune cells to remove necrotic tissue, changes metabolic processes, and induces regeneration mediated simultaneously by cytokines and growth factors within the first five hours after hepatectomy [17]. However, this initiation phase trigger is poorly defined [18]. Hemodynamic changes, activation of the innate immunity, and activation of the Wnt/β catenin and Notch signaling pathways are discussed as major drivers of regeneration induction.

Early hemodynamic alterations in the quantity and quality of portal vein flow have been implicated in beginning the cascade activation. Increased portal volume generates shear stress and the hepatic arterial buffer response reduces the arterial blood flow. Together with activation of the innate immunity, this changes, within 30 min, the concentration of lipopolysaccharides (LPS) in the portal circulation which originate from enteric bacteria and increases the growth factor and cytokine availability for the remaining hepatocytes [19–21] by enhanced release of HGF from the extracellular matrix as well as EGF from Brunner glands [22, 23]. Thereby, nuclear factor KB (NF-KB) becomes free and excites tumor necrosis factor (TNF) and interleukin 6 (IL6) transcription within 30 mins to 1 h after resection [24]. Furthermore, the intrahepatic blood volume and shear stress increases the urokinase plasminogen activator (uPA), activates the extracellular matrix-attached HGF, and increases the activity of HGF- and EGF-activated receptors [25].

Additionally, the pervasiveness of liver sinusoidal endothelial cell (LSEC) fenestrae is enhanced and the secretion of nitric oxide sensitizes hepatocytes to HGF [26]. Quiescent hepatocytes enter the cell cycle and progress from the G0 to the G1 phase of the cell cycle [27]. Two hours after resection, the remaining hepatocytes start to synthesize VEGF, FGF-1 and -2, and angiopoietin-1 and -2 to stimulate the endothelial cells (ECs), PDGF to switch on hepatic stellate cells (HSCs), and TGF-α to act on biliary epithelial cells, and release HB-EGF and amphiregulin (AR). Three hours after resection, new HGFs are produced by the HSCs and ECs.

The proliferative phase starts 5 h after resection and can be divided into a period in which proliferation of hepatocytes and cholangiocytes is induced for 72 h, and an angiogenic phase of 2–3 days in which HSCs, ECs, and Kupffer cells (KCs) proliferate in response to cytokines and growth factors produced by hepatocytes [20].

In the termination phase, autonomic hepatocyte proliferation is restrained by anti-proliferative factors such as transforming growth factor-beta (TGF-β) released from the HSCs and KCs, and activin to ensure normal liver mass and function [28]. However, this important step is not yet well elucidated.

### Potential predictive biomarkers

**Figure 2:**
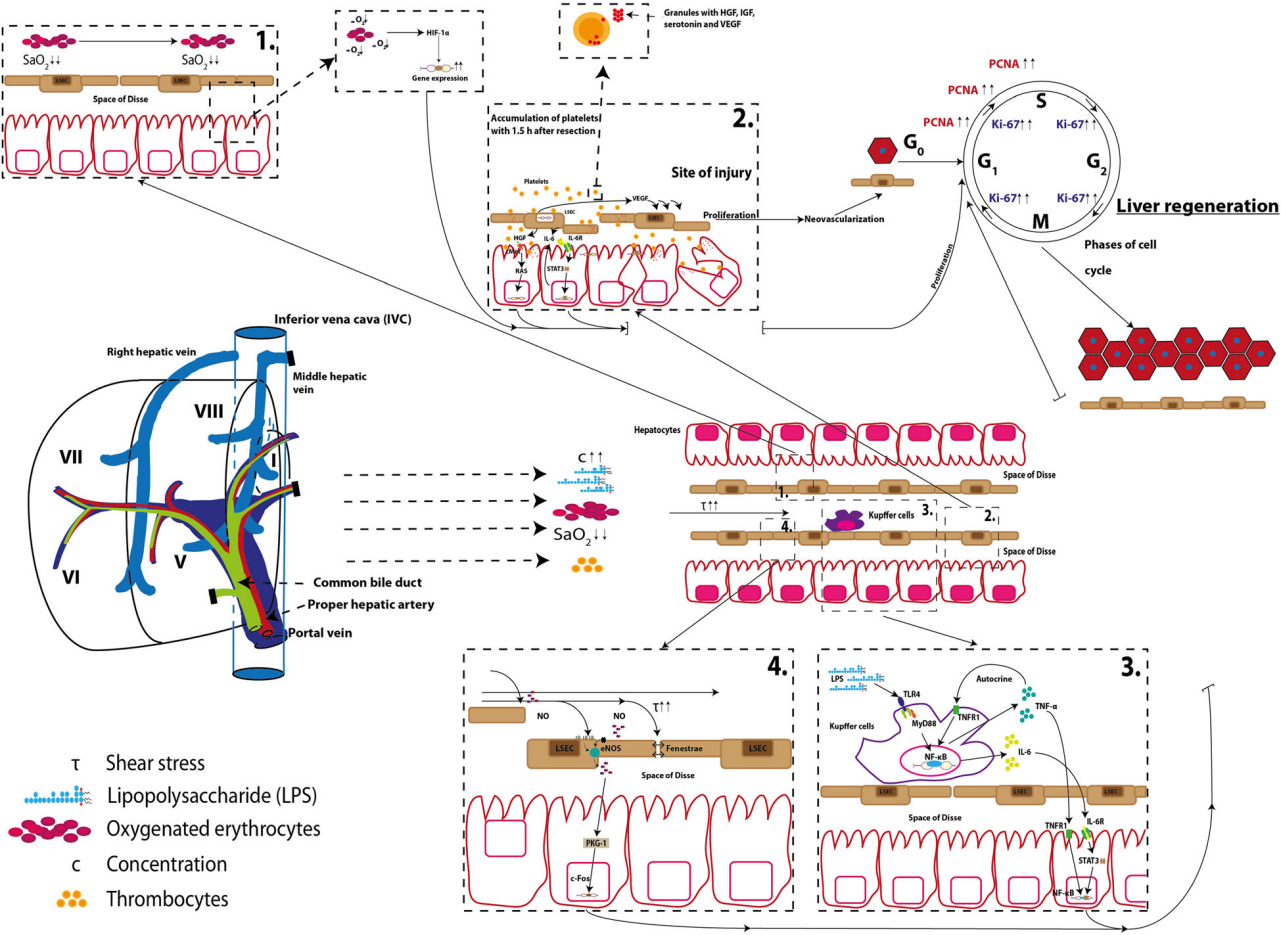
**Fig. 2** Liver regeneration mechanism after resection. 1. Hypoxia via reduced arterial blood flow. 2. Accumulation of platelets and release of growth factors at site of injury. 3. Kupffer-Cell activation via LPS. 4. Activation of regeneration via shear stress

**Figure 3:**
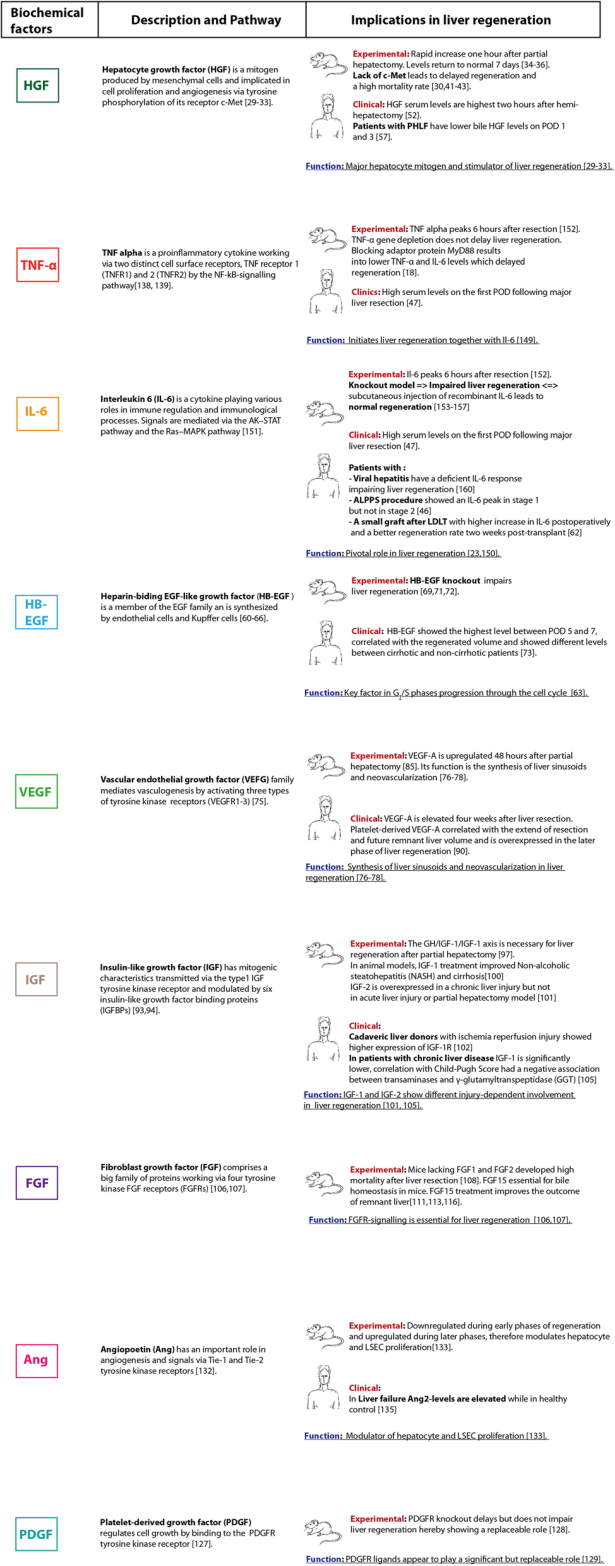
**Fig. 3** Overview of cytokines, growth factors and biological markers involved in liver regeneration

**Figure 4:**
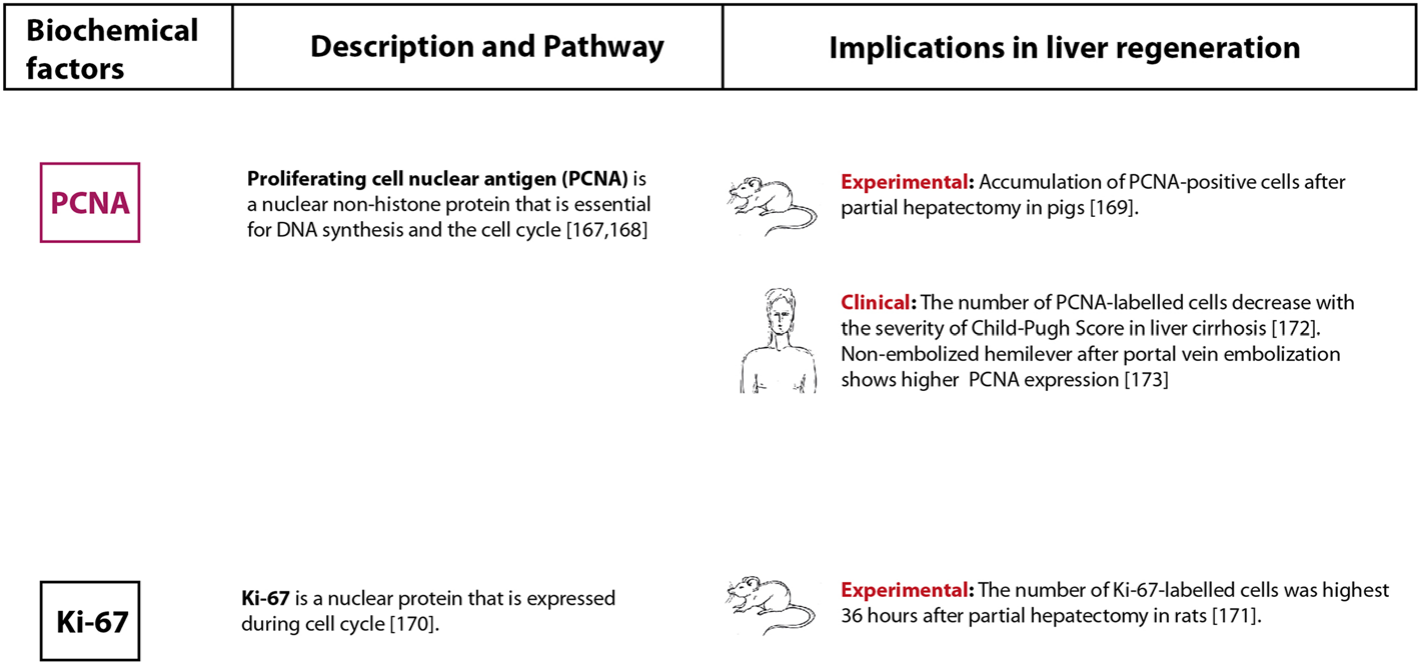
**Fig. 4** Immunhistochemical markers of regeneration

To predict the individual liver regenerative capacity after resection by liver biopsy or preoperative blood samples is an ambitious goal, but offers a great potential to reduce the incidence of PHLF and morbidity as well as mortality rates. The triggers of liver regeneration and modulating cytokines as well as growth factors are closely linked (Fig. 2). In this section, an overview of the key initiators and augmenters during liver regeneration will be provided and the available clinical data on the potential of these factors to predict regeneration capacity will be summarized (Figs. 3 and 4).

### Growth factors

#### Hepatocyte growth factor (HGF)

HGF is a hepatocyte mitogen, originally discovered in 1984, that binds to HGFR/c-MET expressed in parenchymal and non-parenchymal liver cells [29–31]. HGF is synthesized by mesenchymal cells and is attached in an inactivated form to the liver matrix and other organs [32, 33]. In rodents, HGF has been studied intensely. Following a partial hepatectomy, HGF plasma levels increase rapidly (10 to 20 times) to reach concentrations up to 250 ng/ml in rats [34–36]. In the first hours (initiation phase) after a hepatectomy, the increase in HGF originates from existing transcripts of the HGF gene that are localized in the KCs and ECs of normal livers [37]. It is then stimulated in the productive phase by IL-6 and TNF-α triggering from resident immune cells, such as the KCs (hepatic macrophages) that contribute to the immediate response following injury and primarily produce the IL-6 s used for stimulating acute-phase protein production [38, 39]. Later, HGF is newly synthesized by ECs and HSCs. HGF gene expression is also upregulated in the mesenchymal cells of other organs after a liver resection, including the lungs, kidneys, and spleen [40]. Via the HGFR/c-MET receptor, HGF activates the STAT3, PI3K/NF-KB/mTOR, and the RAS/RAF pathways. Data from rodent studies show that a lack of c-MET delays regeneration, leads to liver necrosis and jaundice, and is associated with a high mortality rate [30, 41–43]. A potential use of an exogenously administered HGF activator as an augmenter for liver regeneration was investigated in rats. Recombinant human HGF-activator (rhHGF) was administered via the portal vein and proliferating cell nuclear antigen labelling indices and the liver regeneration rates were significantly higher in the rhHGF-activator group compared to control animals [44].

In humans, HGF, in the context of liver regeneration, has been studied mostly in the setting of living donor liver transplantation and a few studies after resection. All these studies were descriptive and did not analyze a comparable clinical endpoint. However, the HGF levels were elevated after resection on postoperative days (PODs) 1–3 and correlated significantly with the degree of growth of the FLR before stage 2 of the associating liver partition and portal vein ligation for staged hepatectomy (ALPPS) procedure. Stage 1 of the ALPPS procedure begins with transection of the parenchyma along the intended line of resection, and the FLR is cleaned of all tumor tissue in the case of bilobar tumors by partial resection. A temporary portal vein ligation leading to the larger liver lobe is then performed. After a recovery period of 1–2 weeks, Stage 2 is performed in which the deportalized liver is removed to render the patient completely tumor-free [45]. Furthermore, HGF levels were found to be significantly elevated on PODs 1, 7, and 14 after living donor hepatectomy and were correlated with recipient liver volumes on POD 14 [11, 46–55]. Tomiya et al. reported an association of serum HGF levels with hepatocellular dysfunction and systemic inflammation [56]. Takeuchi et al. analyzed bile fluid from percutaneous transhepatic biliary drainage fluid in 24 patients with cholangiocarcinomas undergoing major liver resection and demonstrated that bile, not serum HGF levels on PODs 1 and 3, correlated with the incidence of PHLF. The authors suggested that bile HGF is a potentially useful marker of liver function after liver resection [57]. The outcome difference between HGF serum and bile levels in association with liver regeneration might be explained using the previous finding that 125I-labeled HGF was found to be detectable in the bile and can be excreted from the liver in higher concentrations than in serum [58, 59]. Furthermore, the different serum analysis results are probably due to differences in patient cohorts (cholangiocarcinoma with cholestasis vs. various entities) and sample sizes.

#### Epidermal growth factor (EGF) family

The production of EGF in the Brunner’s glands of the duodenum increases within 30 min after a liver resection and is stimulated by HGF activation, operative trauma associated with the increase of catecholamines from the adrenal glands, release of transforming growth factor α (TGF-α) from hepatocytes 2–3 h after hepatectomy, and heparin-binding EGF (HB-EGF) from KCs and ECs as well as AR within 90 mins after a liver resection. All of these, like EGF, are ligands of the EGF receptor (EGFR) [60–66].

The EGFR is phosphorylated within 60 mins after a hepatectomy and activates via the Ras-Raf-MEK cascade regeneration specific transcription factors (C-myc, C-jun, C-fos), PI3K/AKT/mTOR pathway, and NF-kB system, as well as protein synthesis and cell division via the eukaryotic initiation factor 4E (eIF4E) [20, 22, 41, 42, 67–70]. In rodents, AR and HB-EGF knockout impaired hepatocyte mitosis and led to a delay of liver regeneration and a blockage of EGFRs causing hepatic decompensation. HB-EGF treatment induced protective and regenerative mechanisms following anticholestatic liver injuries [69, 71, 72].

Data on serial measurements of EGF/EGFR ligands in human plasma after surgery in the context of regeneration are extremely rare. Yamada et al. measured serum HB-EGF levels after liver resection and found that the levels were highest between PODs 5 and 7 in patients with major liver resection. Maximal plasma HB-EGF levels correlated significantly with the FLR volume [73]. Tomiya et al. described a significant correlation of TGF-α levels with the resected liver volume and the increased volume of the remaining liver in their analysis of 22 hepatectomized patients with liver cancer. They suggested using serum TGF-α levels as a parameter for evaluating liver regeneration after resection [74]. AR, which is stimulated by acute-phase protein inflammatory signals, has so far only been described in the context of hepatocarcinogenesis and colorectal liver metastases, but not regeneration in humans.

#### Vascular endothelial growth factor (VEGF)

VEGF, FGF-1 and -2, PDEF, and angiopoietin-1 and -2 regulate vascular angiogenesis and restoration of the sinusoidal network during the angiogenic phase of liver regeneration after compensatory hypertrophy. The VEGF family plays a crucial role in regulating vasculogenesis, angiogenesis, and lymphangiogenesis by activating VEGF receptors 1–3 on the surface of endothelial cells of pre-existing blood vessels [75]. VEGF induces the proteolytic activity of matrix metalloproteinases and thereby supports the growth of endothelial cells for formation of new blood vessels as well as the proliferation of ECs, smooth muscle cells, and fibroblasts within the regenerating liver [76–78]. Some animal studies are available [77–83]. VEGF was found to be a central regulator of recruitment for bone marrow progenitors of liver sinusoidal endothelial cells (LSECs) as well as their engraftment in the liver during liver regeneration after resection in rats [84]. VEGF-A, in particular, was found to be upregulated in rat hepatocytes 48 h after partial hepatectomy [85]. Delivery of VEGF-A increased liver masses in mice, but did not stimulate the growth of hepatocytes in vitro, unless the LSECs were also present. Selective activation of VEGFR-1 stimulated hepatocytes, but not endothelial proliferation in vivo, and reduced liver damage in mice exposed to a hepatotoxin [86]. Increases in VEGF receptor Flt-1 in arterioles, sinusoidal ECs in hepatocytes, and Flk-1/KDR in large vessels were detected after 70% partial hepatectomy in rats [87]. VEGFR-1 signaling facilitated liver recovery by reconstitution of sinusoids through recruitment of VEGFR-1-expressing macrophages and by affecting gene expression including hepatotrophic and pro-angiogenic growth factors in mice [88]. Furthermore, VEGFR-2 activity showed a significant increase after partial hepatectomy in transgenic VEGFR-2-luc mice with maximum signals recorded on POD 3 [89]. However, data on humans are sparse. Aryal et al. detected elevated serum VEGF-As and platelet-derived VEGF-As in 37 patients 4 weeks after liver resections. Compared to minor liver resection, platelet-derived VEGF-A levels were higher following major resection and VEGF-A levels correlated with the FLRs [90]. Furthermore, the serum level of soluble VEGFR-2 was a predictive factor for impaired regenerative capacity in humans during the progression from chronic liver disease to liver cirrhosis, but no data were available after resection [91].

#### Insulin-like growth factor (IGF)

IGF factors 1 and 2 mediate growth-promoting mitogenic effects of growth hormones and are involved in the differentiation and inhibition of apoptosis in various cells [92]. Their signals are transmitted through type-1 IGF tyrosine kinase receptors (IGF-1R) mediating both IGF-I and IGF-II signaling, while the type-2 receptor (IGF-2R) decreases the bioavailability of IGF-II. IGF activity is modulated by 6 insulin-like growth factor binding proteins (IGFBPs) [93, 94]. The liver is the main source of circulating IGF-1, synthesized primarily in response to growth hormone. Within the normal adult liver, IGF-II expression is downregulated and IGF-I, although highly expressed, does not exert its actions due to low IGF-IR expression on hepatocytes [95]. However, the role of the IGF-system in the injured liver has not been elucidated. Liver regeneration was found to be delayed in mice lacking the Nrf2 transcription factor because of oxidative stress mediated insulin/IGF-1 resistance that lead to impaired activation of p38 mitogen-activated kinase, Akt kinase, and downstream targets after hepatectomy [96]. Desbois-Mouthon et al. reported that the growth hormone-IGF-1IGF-1R axis was necessary for liver regeneration after partial hepatectomy in liver-specific IGF-IR knockout mice [97]. Targeted over-expression of IGF-1 in activated HSCs accelerated liver regeneration after acute injury and was mediated in part by up-regulation of HGF and downregulation of TGF-β1 [94, 98]. IGF-1 also induces cellular senescence and reduces fibrosis [99]. In animal models, IGF-1 treatment improved non-alcoholic steatohepatitis (NASH) and cirrhosis [100]. IGF-2 is produced by pericentral hepatocytes to promote hepatocyte proliferation and repair tissue damage in the setting of chronic liver injury’; however, this is distinct from the signaling that occurs after resection [101]. Proliferating hepatocytes in rodents responded to IGF-2 through both insulin receptors and IGF-1R. Increased IGF1-receptor expression is reported in hepatocellular carcinoma and patients with chronic hepatitis, which may represent an attempt to stimulate hepatocyte regeneration [102, 103]. Ross et al. demonstrated that key mRNAs involved in the IGF-I axis continue to be expressed in cirrhotic liver despite end-stage liver disease, and therefore, might contribute to the regenerative capacity of the damaged liver [104]. In contrast, Wallek et al. observed significantly lower IGF-1 serum levels in 127 patients with chronic liver disease [105]. However, data on IGF, IGF-1R, or IGFBPs in the context of post-resection regeneration are extremely rare [105]. Stefano et al. observed IGF-1R overexpression in patients receiving cadaveric liver donations 8–12 h after cold ischemia, suggesting that the IGF-1R is involved in liver regeneration [102]. The role of IGF-2 in liver regeneration in humans was investigated by Liu et al. [101]. They concluded that it plays a role in regeneration after chronic injuries like Wilson’s disease, but not in acute recovery after trauma. Based on the sparse information available, additional studies are needed to elucidate the role of IGF-I in human liver regeneration.

#### Fibroblast growth factors (FGFs)

The FGF family is comprised of 22 members in humans and mice with highly different structural characteristics and mechanisms of action. FGF-1 and -2 are produced by hepatocytes [94], and are released by activated HSCs. Together with other growth factors they are responsible for the process of vascular angiogenesis and restoration of sinusoidal networks in the regenerative liver. FGFs transmit signals through 4 tyrosine kinase FGF receptors (FGFRs) and have mitogenic effects in vitro and in vivo [106, 107]. Hepatocyte mitosis is arrested and regeneration was found to be impaired after partial hepatectomy in FGFR-deficient mice [107]. A potential cytoprotective effect of FGF-1 and -2 during liver regeneration was discussed since mice lacking the FGF1R and FGF2R showed impaired cytochrome P450 expression, liver failure, and increased mortality after liver resection [108]. The treatment of primary hepatocytes isolated from the regenerating liver with the FGF-7 protein activated ERK1/2 and promoted proliferation [109]. FGF-19 and FGF-21 promote important hepatoprotective activities and, in the light of promising mouse experiments, are considered to have a potential application for the clinical management of acute liver injuries [110]. After liver resection, a rapid but transient bile acid overload in the liver leads to the first wave of proliferative signaling in the remnant hepatocytes. Bile acids trigger hepatocyte proliferation through activation of several nuclear receptors. Following biliary passage into the intestines, enterocytes reabsorb the bile acids, which result in the activation of farnesoid X receptor (FXR) and excretion of FGF-19/FGF-15 and its release into the enterohepatic circulation. FGF-15, a bile-acid-induced ileum-derived enterokine, was found to be essential for bile acid homeostasis and was identified as an essential mediator of the liver growth-promoting effects of bile acids during liver regeneration in mice [111–113]. This is interesting since regeneration is impaired in cholestatic liver as well as in liver with interrupted bile acid provision through enterohepatic circulation, e.g., by external biliary drainage [112, 114]. Padrissa-Altés et al. demonstrated that the FGF-15/FGFR-4/STAT-3/Fox-M1 axis controls hepatocyte proliferation and that loss of FGF-R1, −R2, and -R4 evokes liver failure after partial hepatectomy [115]. Recently, the FXR agonists have been shown to promote regeneration via the gut-liver axis and might be beneficial for patients with hepatobiliary tumors undergoing resection [116]. Data on the effects of FGFs after resection in humans are extremely rare and no recommendations for their use as biomarkers can be provided.

#### Platelet-derived growth factor (PDGF)

In humans, low preoperative platelet counts correlates with higher PHLF rates and higher mortality after hepatectomy [117]. Platelets accumulate within the initiation phase of regeneration at the resection surface, are critical modulators of tissue repair, and contain granules of HGF, serotonin, VEGF, and IGF [118, 119]. Platelets are potent inducers of liver regeneration after partial hepatectomy and platelet activation as well as granule release increase after liver resection [120, 121]. Platelets adhere to LSECs and hepatocytes and induce the proliferation of these cells [77, 122, 123]. Furthermore, they synthesize and store PDGFs [124], which switch on HSCs, enhances their growth, and propagates signaling (e.g., TGF-β1). Together with their ligands, they regulate cell growth and angiogenesis [91, 125] producing new mature well-stabilized blood vessels. PDGFs are stored in α-granules and released during the very early stages of liver regeneration [126]. Furthermore, their release from activated hepatocytes 2–5 h after partial hepatectomy has been demonstrated [25]. PDGF-A and -B undergo intracellular activation during transport in the exocytic pathway for subsequent secretion, whereas PDGF-C and -D are secreted as latent forms that require activation by extracellular proteases. PDGFs bind to the tyrosine kinase receptors, PDGFR-α and PDGFR-β [127]. High levels of PDGFR-α expression were detected 3 h after partial hepatectomy in mice. In contrast, PDGFR-α knockout mice showed impaired PDGF signal transduction that compromised extracellular signal-regulated kinases and AKT (a serine/threonine-specific protein kinase) activation. However, PDGF is alleviated by temporal compensatory increases in the expression and activation of EGFR and HGFR along with rebound activation of extracellular signal-regulated kinases and AKT at 24 h [128]. These results attest to the signaling ‘flexibility’ that is a well-recognized theme in liver regeneration. Similar to most growth factors in liver regeneration following a liver resection, ligands of PDGFR-α appear to play a significant, but replaceable role [129].

The hepatic expression of all PDGF isoforms and receptors at both mRNA and protein levels increased in rats after acute liver injury, peaked at 4 weeks, and decreased thereafter to near basal levels after 8 and 12 weeks [130]. Conditional PDGFR-β deletion in HSCs led to disrupted PDGF signaling with prolonged liver injury in rodents. However, the overall regeneration capacity was not affected. The role of PDGFs in liver regeneration in humans has not been fully analyzed [131]. Starlinger et al. demonstrated that the profile of the α-granule content released from the platelets affects the postoperative outcome. They provided evidence that increased postoperative portal venous pressure is associated with an unfavorable α-granule release profile (high thrombospondin 1/low VEGF). In their analysis of 157 patients undergoing liver resection, morbidity and prolonged hospitalization were associated with this unfavorable protein profile. However, further studies are warranted to elucidate the role of PDGFs as markers for liver regeneration.

#### Angiopoietin (Ang)

After exposure of the liver to injurious events, angiopoietins are produced by hepatocytes. Together with other factors, Ang-1 and -2 are responsible for vascular angiogenesis and restoration of sinusoidal networks via duplicating hepatic endothelial cells. They transmit signals via the Tie-1 and -2 tyrosine kinase receptors [132]. Ang-2 dynamically modulates liver regeneration by orchestrating hepatocyte and LSEC proliferation. The expression is downregulated in the LSECs during the early phase of post-hepatectomy liver regeneration and recovers in the later phases [133]. During the early phase, Ang-2 downregulation leads to hepatocyte proliferation by reduced LSEC TGF-β1 production and enhanced expression of cyclin D1 in a paracrine manner. In contrast, in the recovery phase, it enables non-parenchymal cell regeneration and angiogenesis in an autocrine manner by controlling LSEC VEGFR-2 expression and Wnt-2 signaling [134].

Ang-2 levels increased in liver biopsy samples of 37 patients with primary acute liver failure, regardless of their etiology or liver dysfunction status, while it was almost absent in a healthy control group [135]. Data regarding Ang-2 expression after liver resection are not valid for regeneration since they were also obtained in HCC patients who had varying Ang-2 expression within the tumors [136].

### Cytokines

Cytokines are pleiotropic regulatory peptides that are produced in most types of liver cells [137]. Constitutive production is minimal, but upon physiologic or pathologic stimulation, the key regulators, TNF-α and IL-6, mediate hepatic inflammation, apoptosis, and necrosis of damaged liver cells, and also mediate the regeneration of liver tissue after injuries.

#### Tumor necrosis factor alpha (TNF-α)

TNF-α is a proinflammatory cytokine that belongs to the TNF superfamily and stimulates the synthesis of acute-phase proteins. It activates the NFκB signaling pathway directly via binding on the TNF receptor 1 (TNF-R1) on KCs and indirectly through induction of the inhibitory KB kinase [138, 139]. Furthermore, it activates hepatocyte proliferation through stimulation of c-Jun N-terminal kinase, phosphorylation of c-Jun-transcription-factor in the nucleus, and induction of target gene transcription, such as cell division cycle protein 2 homolog (CDC2/CDK-1) [22, 140]. Hepatic macrophages (KCs) are the main source of TNF-α triggered either by gut-derived factor lipopolysaccharide (LPS)/Toll-like receptor 4 (TLR4) signaling, or by C3a and C5a components of the complement system. TNF-α was found to sensitize hepatocytes to growth factors in a rat partial-hepatectomy model [141]. Its gene expression is upregulated 30–120 min after hepatectomy [142, 143]. TNF-α and Il-6 induction requires the adaptor protein MyD88. In mice lacking this protein, the TNF-α and Il-6 levels were lower after partial hepatectomy and liver regeneration was slower [18]. TNF-α also promotes KC functions via autocrine stimulation and boosts their activation [144]. However, complete deletion of the TNF-α-gene did not delay regeneration which indicates that TNF-α is not involved in the later stages of regeneration [47, 145]. In humans, the role of TNF-α has been investigated in the context of liver graft regeneration after living donor liver transplantation. Sasturkar et al. investigated 25 patients undergoing right donor lobe hepatectomy and reported significantly higher TNF-α in their sera on POD 1 compared with baseline measurements [47]. Furthermore, a correlation of higher preoperative serum levels of TNF-α with increased relative liver volumes at POD 7 was reported. Serial measurements of TNF-α before and after hepatic resection detected only slight elevations, but no correlations with hepatic regeneration [146]. Based on those data, the monitoring of regeneration by TNF-α cannot be recommended [147].

#### Interleukin 6 (IL-6)

IL-6 is secreted during inflammatory conditions upon LPS stimulation in a TNF-*α-*dependent/−independent manner [148, 149]. In response to liver injury, IL-6 mediates the acute-phase response and induces both cytoprotective and mitogenic functions. It is a critical component in priming the hepatocytes for proliferation being responsible for the activations of approximately 40 genes which are not expressed in the normal liver, but which are immediately triggered in remaining liver tissue after partial hepatectomy [23, 150].

Signals are mediated via the Janus family tyrosine kinase/signal transducer and activator of transcription (JAK–STAT) pathway and the Ras–MAPK pathway [151]. Circulating IL-6 s peak within 6 h after liver resection [152]. Cressmann et al., demonstrated that IL-6 gene disruption impairs liver generation in mice. In contrast, introducing IL-6 enabled hepatocyte proliferation by activating the STAT3 pathway [153, 154]. This was confirmed since injecting recombinant human IL-6 (1 mg/kg) into TNFR-I-deficient animals 30 min before partial hepatectomy restored the initial STAT3 binding deficiency [155]. Blindenbacher et al., showed that a subcutaneous injection of recombinant human IL-6 (500 ng/g) prevented postoperative mortality in knockout mice as long as the injections were sustained [156]. IL-6-induced activation of STAT3 boosted hepatic gene expression to maintain metabolic homeostasis after liver resection [157].

In humans, a peak in the IL-6 levels within 6 h after resection that was associated with the remnant liver volume was detected, which slowly decreased over the following days [158]. Serial measurements of IL-6 levels after partial hepatectomy revealed that the levels of IL-6 increased immediately after the operation. IL-6 is considered to be a sensitive marker of surgical stress, induction of hepatic regeneration, and the production of acute phase proteins in the liver [146]. The levels of IL-6 were found to be significantly lower in the hepatic vein compared to the radial artery and the portal vein at the end of the resection. The authors concluded that circulating IL-6 s might be taken up and used in the liver and suggested monitoring the difference between arterial and hepatic venous blood levels as an indicator for regeneration [159]. Furthermore, deficient IL-6 responses were considered to be a major cause of impaired regeneration after hepatectomy in patients with viral hepatitis [160]. Measurements of IL-6/HGF ratios in the local exudative fluid after hepatectomy suggested that both proteins are produced at the site of injury, but HGF may predominate [161]. ALPPS procedures resulted in a peak of IL-6 levels after stage 1, which decreased rapidly and did not increase after stage 2. Furthermore, a correlation between the peak IL-6 levels and HGF was detected [46]. In the setting of human living donor liver transplantation, higher levels of serum IL-6 were independently associated with increased graft volumes during the first postoperative week [147]. Oyama et al. demonstrated that patients with a small graft after living donor liver transplantation showed a higher increase in IL-6 levels postoperatively and a better regeneration rate 2 weeks post-transplant [162]. A potential use of exogenously administered recombinant IL-6 (rhIL-6) as an inducer of regeneration was investigated in a pilot study by de Jong et al. [163]. RhIL-6 administration resulted in an increase of serum HGF, but its effects on the liver were not evaluated.

### Immunohistochemical evaluation

In animal models, liver regeneration is monitored by histological evaluation of liver tissue [164]. The most common method is staining proliferating cells [165] which tracks cell growth and division with proliferation markers (Fig. 4). In humans, a rapid and inexpensive approach to monitor regeneration might be analysis of liver biopsy samples, PCNA, or Ki-67.

#### PCNA and Ki-67

PCNA and Ki-67 are markers of cell proliferation routinely used in clinical pathology [166]. PCNA is a nuclear non-histone protein that is essential for DNA synthesis during the cell cycle. It also plays a role in DNA replication and repair. PCNA expression is elevated during the late G1 to S phase of the cell cycle. Quiescent and senescent cells have very low levels of PCNA mRNA [167, 168]. Moreover, Nygård et al. showed a gradual accumulation of PCNA-positive cells in the periportal region 6 weeks after 60% partial hepatectomy in pigs. This supported the ‘streaming hypothesis’, which states that the newly generated hepatocytes migrate from the periportal to the central region [169].

The protein Ki-67 is present in the cell nucleus during the late G1, S, G2, and M phases of the cell cycle. It is absent in resting cells (G0) [170]. The highest number of Ki-67 labelled cells was detected 36 h after partial hepatectomy in rats. Labelled cells were located primarily periportally [171]. Data on humans are again rare. Delhaye et al. observed that the indices of PCNA labelled cells decreased with increasing Child-Pugh scores in patients with liver cirrhosis. After transjugular intrahepatic portosystemic shunts, the indices dropped significantly further suggesting that reduced blood flow impairs regeneration [172]. This was confirmed by Harada et al., who detected a low PCNA expression in the hemi-liver after portal vein embolization before an extended right lobectomy while high PCNA expression was observed in the non-embolized portion. The authors concluded that PCNA is an indicator of hepatocyte proliferation and liver growth [173]. However, histological evaluation of liver regeneration by biopsy must be discussed in a controversial setting. Since liver regeneration occurs over a course of many weeks, regular biopsy would be necessary to monitor the process. This implies that patients with reduced liver function after resection are prone to serious clinical problems, particularly, coagulopathy [174, 175].

#### Circulating microRNAs (miRNAs)

In additional to the above mentioned markers, there is emerging evidence that miRNAs might represent prognostic biomarkers for liver regeneration [176]. Various miRNAs regulate liver functions and miR-122 in particular was identified to play a role in regulating liver function in a variety of liver diseases [177]. An HGF dependent increase of levels of miRNA expression was detected in vitro linking the classical cytokine and growth factor induced regeneration pathways with miRNAs as key regulators of various biological processes in the liver [178]. Experiments in rodents revealed that miR-122 is an early and sensitive biomarker of hepatocellular injury at a stage when alanine transaminase, aspartate transaminase, and total bilirubin are not detectable. Furthermore, time-course changes in the expression levels have been shown [179]. An increasing number of studies have investigated circulating miRNAs regarding their prognostic potential for acute liver injury. John et al., showed that miR-122, miR-21, and miR-221 are involved in liver regeneration and might contribute to spontaneous recovery from acute liver failure [180]. Furthermore, miR-194, miR-210, miR-483, miR-4532, and miR-455-3p were identified as diagnostic biomarkers in acute liver failure [181–183]. In a small cohort of patients, Starlinger et al. identified the miRNA signature, which consisted of circulating miRNAs 151a-5p, 192-5p, and 122-5p, as a potential prognostic tool for predicting postoperative liver dysfunction, morbidity, and even mortality. Furthermore, the authors detected dynamic changes in miRNA expression in the perioperative course [184]. However, confirmatory studies with larger patient cohorts are needed to provide evidence for whether miRNA profiling may represent an improved strategy to identify patients at high risk for liver failure.

## Discussion

The liver’s regenerative potential is legendary and depends on a carefully orchestrated symphony of factors that enable a precise and timely recovery of the liver’s metabolic and synthetic functions after resection. The critical time frame for regaining hepatic function and successful recovery after partial hepatectomy appears to be 5–7 days. However, prediction of the individual regenerative capacity with the goal of promoting hepatic regeneration in our most gravely ill patients is still emerging. The available data for monitoring and predicting PHLF in humans, based on growth factor and cytokine expression, are highly heterogenic, with most of these data obtained from observational studies. Typically, the case numbers are low, and clinical setting includes resection as well as transplantation; the analyzed blood and tissue samples were collected at various time points, and the described endpoints were extremely variable. The goal to find a single marker that accurately predicts liver regeneration in liquid biopsy samples had to be abandoned with regard to overlapping and partly redundant pathways. To address the heterogeneity of patients and the large numbers of potential markers, high throughput serial analyses would be helpful to screen, validate, and confirm biomarkers that predict regenerative potential.

## Conclusions

High level evidence on serial measurements of growth factors and cytokines in blood samples used to predict liver regeneration after resection is lacking. Some promising marker candidates for peri-operative monitoring might be HGF, IL-6, and VEGF. To promote their confirmation, large-scale, multi-center prospective clinical trials are required. However, profiling their individual regenerative capacity after liver resection is not yet possible.

## Data Availability

Not applicable.

## Abbreviations

ALPPS: Associating liver partition and portal vein ligation for staged hepatectomy
Ang: Angiopoietin
AR: Amphiregulin
CDC2: Cell division cycle protein 2
ECs: Endothelial cells
EGF: Epidermal growth factor
EGFR: EGF receptor
eIF4E: Eukaryotic initiation factor 4E
ERK1/2: Extracellular signal-regulated kinase 1/2
FGF: Fibroblast growth factor
FGFR: FGF receptor
Flk-1/KDR: Fetal liver kinase 1/Kinase insert domain receptor
FLR: Future liver remnant
FXR: Farnesoid X receptor
HB-EGF: Heparin-binding EGF
HCC: Hepatocellular carcinoma
HGF: Hepatocyte growth factor
HGFR: Hepatocyte growth factor receptor
HSCs: Hepatic stellate cells
IGF: Insulin-like growth factor
IGF-R: Insulin-like growth factor receptor
IL6: Interleukin 6
INR: International normalized ratio
KC: Kupffer cell
LPS: Lipopolysaccharides
LSEC: Liver sinusoidal endothelial cell
MAPK: Mitogen-activated-protein kinase
miRNA: microRNA
mRNA: Messenger RNA
mTOR: Mechanistic target of Rapamycin
NASH: Non-alcoholic steatohepatitis
NF-KB: Nuclear factor kappa-light-chain-enhancer of activated B-cells
PCNA: Proliferating cell nuclear antigen
PCR: Polymerase chain reaction
PDGF: Platelet-derived growth factor
PDGFR: Platelet-derived growth factor receptor
PHLF: Post-hepatectomy liver failure
PI3K: Phospoinositid-3-Kinase
PICOS strategy: Population, Intervention, Comparison, Outcome and Study design strategy
POD: Postoperative day
RAF: Rapidly accelerated fibrosarcoma protein
RAS: Rat sarcoma Proto-Onkogen
STAT3: Signal transducer and activator of transcription 3
TGF-alpha: Transforming growth factor alpha
TGF-β: Transforming growth factor beta
TLR4: Toll-like receptor 4
TNFR1: Tumor necrosis factor receptor 1
TNF-α: Tumor necrosis factor alpha
uPA: Urokinase plasminogen activator
VEGF: Vascular endothelial growth factor
VEGFR: Vascular endothelial growth factor receptor
